# Tradeoffs in milk immunity affect infant infectious disease risk

**DOI:** 10.1093/emph/eoac020

**Published:** 2022-06-13

**Authors:** Katherine Wander, Masako Fujita, Siobhan M Mattison, Margaret Duris, Megan Gauck, Tessa Hopt, Katherine Lacy, Angela Foligno, Rebecca Ulloa, Connor Dodge, Frida Mowo, Ireen Kiwelu, Blandina T Mmbaga

**Affiliations:** Department of Anthropology, Binghamton University (SUNY), Binghamton, NY, USA; Department of Anthropology, Michigan State University, East Lansing, MI, USA; Department of Anthropology, University of New Mexico, Albuquerque, NM, USA; Department of Anthropology, Binghamton University (SUNY), Binghamton, NY, USA; Department of Anthropology, Binghamton University (SUNY), Binghamton, NY, USA; Department of Anthropology, Binghamton University (SUNY), Binghamton, NY, USA; Department of Anthropology, Binghamton University (SUNY), Binghamton, NY, USA; Department of Anthropology, Binghamton University (SUNY), Binghamton, NY, USA; Department of Anthropology, Binghamton University (SUNY), Binghamton, NY, USA; Department of Anthropology, Binghamton University (SUNY), Binghamton, NY, USA; Kilimanjaro Christian Medical Centre, Moshi, Kilimanjaro, Tanzania; Kilimanjaro Christian Medical University College, Moshi, Kilimanjaro, Tanzania; Kilimanjaro Christian Medical Centre, Moshi, Kilimanjaro, Tanzania; Kilimanjaro Christian Medical University College, Moshi, Kilimanjaro, Tanzania; Kilimanjaro Clinical Research Institute, Moshi, Kilimanjaro, Tanzania; Kilimanjaro Christian Medical Centre, Moshi, Kilimanjaro, Tanzania; Kilimanjaro Christian Medical University College, Moshi, Kilimanjaro, Tanzania; Kilimanjaro Clinical Research Institute, Moshi, Kilimanjaro, Tanzania; Duke Global Health Institute, Duke University, Durham, NC, USA

**Keywords:** maternal and child health, lactation and breastfeeding, immune system of milk, pneumonia and respiratory infection

## Abstract

**Background and objectives:**

The human immune system has evolved to balance protection against infection with control of immune-mediated damage and tolerance of commensal microbes. Such tradeoffs between protection and harm almost certainly extend to the immune system of milk.

**Methodology:**

Among breastfeeding mother–infant dyads in Kilimanjaro, Tanzania, we characterized *in vitro* proinflammatory milk immune responses to *Salmonella enterica* (an infectious agent) and *Escherichia coli* (a benign target) as the increase in interleukin-6 after 24 h of incubation with each bacterium. We characterized incident infectious diseases among infants through passive monitoring. We used Cox proportional hazards models to describe associations between milk immune activity and infant infectious disease.

**Results:**

Among infants, risk for respiratory infections declined with increasing milk *in vitro* proinflammatory response to *S. enterica* (hazard ratio [HR]: 0.68; 95% confidence interval [CI]: 0.54, 0.86; *P*: 0.001), while risk for gastrointestinal infections increased with increasing milk *in vitro* proinflammatory response to *E. coli* (HR: 1.44; 95% CI: 1.05, 1.99; *P*: 0.022). Milk proinflammatory responses to *S. enterica* and *E. coli* were positively correlated (Spearman’s rho: 0.60; *P*: 0.000).

**Conclusions and implications:**

These findings demonstrate a tradeoff in milk immune activity: the benefits of appropriate proinflammatory activity come at the hazard of misdirected proinflammatory activity. This tradeoff is likely to affect infant health in complex ways, depending on prevailing infectious disease conditions. How mother–infant dyads optimize proinflammatory milk immune activity should be a central question in future ecological–evolutionary studies of the immune system of milk.

## BACKGROUND 

In addition to supporting infant nutrition and development, human lactation and breastfeeding have evolved to protect infants against infectious diseases (ID): breastfed infants have dramatically lower ID risk than infants fed milk substitutes [1–4]. The activity of the immune system of milk (ISOM) likely plays an important role in this protection; however, the extent to which this activity is subject to well-characterized tradeoffs between protection against infection and immune-mediated damage remains incompletely understood. Indeed, the extent and complexity of milk immunity has only recently been described, and the impact of the ISOM on infant ID risk is remarkably understudied, particularly given the relevance of evolutionary biology for understanding these tradeoffs and their implications for lifelong health.

The ISOM includes leukocytes, antibodies, cytokines (immune cell communication molecules) and antimicrobial proteins that likely play important roles in defense against ID and infant immune system development [5–8] by creating an immune axis within the breastfeeding dyad. Antibodies, antimicrobial proteins and immune cells from milk can act directly against infectious agents in the intestinal lumen [9]; ISOM factors can assist in coordinating systemic immune responses via infant gut-associated lymphoid tissue [10]; and, the ISOM can transfer lasting immunological memory of infectious agents when milk leukocytes move to the infant thymus and train maturing leukocytes [11]. Thus, it seems likely that this mother–infant immune axis evolved to protect infants against ID.

While the importance of breastfeeding in protecting against ID is quite clear when breastfed infants are compared to those who receive formula, substantially less research links milk immune content or activity to infants’ ID risk using comparisons among breastfeeding mother–infant dyads. This is despite the observation that milk immune content is highly variable across mother–infant dyads [12]. Early research connected milk pathogen-specific secretory immunoglobulin A (sIgA) to lower risk for *Campylobacter* infection and lower risk for symptoms upon infection with *Shigella* or *Giardia* among Mexican infants [13–15]. Recent research has further demonstrated an inverse association between milk total sIgA and subsequent diarrheal disease symptoms among Qom (https://www.carepercha.com/) infants in Argentina [16]. In addition, a positive association between milk anti-inflammatory factors and infant length-for-age in Kenya may suggest that these factors in milk promote infant growth [17]. Together, these studies suggest that variation in the ISOM is an important factor affecting infant ID risk and health, with delivery of larger quantities of immune factors via milk conferring a protective benefit to infants.

A central challenge of immune system evolution—balancing the multifactorial costs and benefits of immune activity—almost certainly pertains to the ISOM and the mother–infant immune axis it creates. Costs of immune activity include collateral damage to tissues caused in the course of combating infectious agents [18]; ‘over’-responses to infectious agents that can be life-threatening [19–21]; misdirected immune responses against otherwise innocuous stimuli that underlie allergic and autoimmune disease [22]; and, immune-mediated damage to beneficial microbes that may contribute to chronic disease etiology [23, 24]. These costs to immune activity occur because many immune mechanisms, such as inflammation, cannot be perfectly targeted to affect only infectious agents or infected host cells [25]. In addition, both immune activity and immunotolerance are energetically expensive [26], and evolved mechanisms that optimize energy allocation may not minimize disease risk [27–29].

ISOM activity may have particularly high costs and benefits for the mother–infant dyad, both because infancy is a period of high vulnerability to ID, and because ISOM activity may affect not only infants’ short-term survival, but also, through effects on immune system development, their future health and fitness. Thus, we may expect infants to experience tradeoffs between ISOM-mediated protection and harm across short-term timescales. We may also expect, over longer timescales, that the ISOM shapes infant immune system development to manage these tradeoffs, by promoting robust immune regulation, facilitating the development of a healthy microbial flora and lowering infants’ risk for immune-mediated damage and disease.

We sought to employ an evolutionary optimization framework and a focus on tradeoffs in milk immune activity to understand how the ISOM impacts infant health across short-term timescales. To this end, we assessed milk immune content, employed a newly developed method to assess ISOM activity in the presence of harmful and benign targets, and evaluated how milk immune content and activity predicted infant ID risk.

We characterized milk immune content by estimating concentrations of sIgA and the cytokines interleukin-6 (IL-6), interleukin-10 (IL-10) and interferon-γ (IFN-γ). sIgA is the dominant antibody found in milk [12] and is often evaluated as a biomarker of milk immune value or the protective capacity of milk [16, 30]. IL-6 is a proinflammatory cytokine produced by leukocytes to upregulate immune responses [31, 32], while IL-10 is a generally anti-inflammatory cytokine [33, 34] and IFN-γ promotes the development of T-helper type 1 adaptive immune responses [35].

To characterize milk’s capacity for immune **activity**, we assessed cytokine (IL-6, IL-10 and IFN-γ) responses to *in vitro* stimulation with two bacteria, *Salmonella enterica* and *Escherichia coli* [36]. *Salmonellaenterica* causes a variety of diarrheal diseases of global public health importance. *Escherichiacoli* is a normal constituent of the human gut; most strains are benign or beneficial, although some strains are pathogenic to humans. This technique was developed to go beyond description of the ISOM by its constituent parts to provide a **system-level** characterization of ISOM activity, favoring organism-level to cellular- or molecular-level information [36].

We evaluated these measures of milk immune content and activity as predictors of infant ID risk among mother–infant dyads in rural Kilimanjaro, Tanzania, a setting of particularly high ID risk in infancy [37]. We expected the capacity of the ISOM to respond to pathogenic targets to protect infants against ID, and predicted lower incidence of ID among infants receiving milk that showed higher levels of *in vitro* activity when stimulated with *S. enterica*. We further anticipated that ISOM responses to benign targets, captured by higher levels of *in vitro* activity when stimulated with *E. coli*, might have adverse effects on infants’ health.

## MATERIALS AND METHODS

### Setting and participants

Data were collected in Kilimanjaro, Tanzania, in 2019. Approximately, 100 singleton infants 0–12 months of age (on the day of sample selection) were randomly selected from among participants in a Maternal and Child Health (MCH) program in rural Kilimanjaro and their mothers were invited to participate. Mothers who were HIV positive or not lactating were excluded. Data collection and field laboratory work were completed in the health clinic hosting the MCH program.

### Informed consent and oversight

Written informed consent was obtained by study staff from all adult participants. Ethical review and oversight were provided by Kilimanjaro Christian Medical University College (819) and the United Republic of Tanzania National Institute for Medical Research (NIMR/HQ/R.8a/Vol IX/2366). Binghamton University’s Institutional Review Board relied on the findings and oversight of Kilimanjaro Christian Medical University College. Research clearance for non-Tanzanian team members was obtained from the United Republic of Tanzania Commission on Science and Technology National Research Registration Committee.

### Questionnaire

Mothers provided their date of birth and information about their health and reproductive histories, breastfeeding practices, household composition, and their infant’s recent health via questionnaire at the initial participation visit. Infants’ estimated due date, birthweight, and mode of delivery were obtained from birth records.

### Anthropometry

Maternal height was measured with a portable stadiometer (Seca 213) and weight with a digital scale (Seca 876). Infant length was measured with a measurement mat (Seca 210) and weight with a digital infant scale (Seca 334).

### Infectious disease monitoring

Mothers were invited to return to the clinic after their initial visit in the event of infant illness during the duration of the project (∼2.5 months). Physicians provided care, including physical exams and screening for malaria via rapid diagnostic test, and diagnosis and treatment were recorded.

### Specimen collection

One drop of whole blood was collected via finger stick from each mother and tested for HIV with a rapid diagnostic test (SD Bioline HIV-1/2) to verify eligibility.

Mothers wiped the skin of the breast used least recently for infant feeding with a ‘hand sanitizer’ wipe (Purell), then expressed ∼10–30 ml of milk with a manual pump (Lansinoh) into clean and heat sterilized bottles. Aliquots of milk were isolated and immediately used for *in vitro* stimulation (below). Additional 1 ml aliquots of whole milk specimen were isolated and frozen in the field laboratory inside the clinic (−20°C) until transfer to the Kilimanjaro Clinical Research Institute (KCRI), where they were frozen (−80°C) until transfer via courier on dry ice to the Laboratory for Anthropometry and Biomarkers (LAB) at Binghamton University (SUNY), where specimens were thawed and aqueous fractions were isolated via centrifugation and frozen (−80°C).

### *In vitro* whole milk stimulation

One lyophilized pellet of *S. enterica* (Microbiologics 0363 L; ATCC 14028) or *E. coli* (Microbiologics 0791E3; ATCC 51813) was combined with 1 ml purified (Type II) water, then diluted to 2% in RPMI 1640 mammalian cell culture medium (Lonza 12918F) prepared with 0.11 mg/ml sodium pyruvate (Lonza 13115E), 0.292 mg/ml L-glutamine (HyClone SH30034) and 100 U/ml penicillin–streptomycin (Gibco 15140148), to form *S. enterica* and *E. coli* stimulants.

In the field laboratory, immediately after expression, 2 ml of milk was combined with 1 ml of stimulant in one well of a 6-well suspension plate (one well each for *S. enterica* or *E. coli*). Plates were covered and placed in a sealed acrylic container with a small dish of water and a lit candle to create a humid, CO_2_-rich environment. The candle was allowed to extinguish in the sealed container and then the container was placed in a 37°C incubator for ∼24 h. Additional details and validation of the *in vitro* whole milk stimulation protocol are provided in [36].

After incubation, specimens were frozen in the field laboratory (−20°C) until transfer to the KCRI laboratory, where they were frozen (−80°C) until transfer via courier on dry ice to the LAB, where specimens were thawed and aqueous fractions were isolated via centrifugation and frozen (−80°C).

### Enzyme immunoassay

All enzyme immunoassay (EIA) analyses were performed in the LAB following kit instructions or published methods. Cytokine (IL-6, IL-10 and IFN-γ) concentrations were estimated in baseline (unstimulated) and stimulated specimens using the Quansys Biosciences 4-plex High Sensitivity Human Cytokine (112549HU) kit. Intra-assay coefficients of variation (CVs) across the 14 plates included in these analyses were: IL-6: 7.4%; IL-10: 10.6%; IFN-γ: 8.6%. Inter-assay CVs were assessed from values estimated for the kit calibrators (mean for 7 points) in lieu of assay controls: IL-6: 11.2%; IL-10: 13.8%; IFN-γ: 16.7%. Concentrations of sIgA were estimated in baseline specimens with an in-house assay following Hassiotou and colleagues [38]; the inter-assay CV was 5.2% and the intra-assay CV was 6.2% for the six plates evaluated here. EIA estimated concentrations were corrected for assay dilution, and incubated specimens were further corrected with a factor of 1.5 for dilution prior to incubation in RPMI culture medium.

To deal with inestimably low cytokine concentrations, for each cytokine, the value of the highest lower limit of detection (LLOD) across all assay runs, multiplied by the correction factors of 1.5 for dilution at the time of stimulation and 2.0 for dilution at the time of assay, was considered the global ‘low’ value for that cytokine; all inestimably low specimens, as well as those estimated to have a concentration below this value, were assigned the global low [36]. The global low was assigned to 76 baseline specimens, 18 *S. enterica-*stimulated specimens and 19 *E. coli*-stimulated specimens for IL-6; all baseline and *E. coli*-stimulated specimens and 91 *S. enterica-*stimulated specimens for IL-10; and, 95 baseline specimens, 95 *S. enterica*-stimulated specimens and 83 *E. coli-*stimulated specimens for IFN- γ. sIgA concentrations were estimable for all specimens.

### Data analysis

Cytokine responses to *S. enterica* and *E. coli* were defined as the ratio of cytokine concentrations in the stimulated specimen to the baseline specimen: [stimulated]/[baseline] (e.g. an IL-6 response of 4 reflects a four-fold increase in IL-6 after 24 h incubation with *S. enterica* or *E. coli*).

Z-scores for infants’ weight-for-length and length-for-age were calculated with Stata SE 15.0 software using the WHO 2006 child growth standards [39].

We considered only incident ID (those that were diagnosed during sick visits made subsequent to the initial participation visit) in these analyses. Recorded diagnoses were used to categorize episodes as upper respiratory tract infection (URTI), bronchitis, pneumonia, gastrointestinal infection and other ID. Non-ID diagnoses (e.g. allergy, injury) were ignored. When two ID were diagnosed at the same visit, the infant was considered an incident case within each category. Only the first diagnosis within each ID category was captured; if an infant received a subsequent diagnosis within a category, this was ignored in analyses.

We used Spearman’s rank-order correlation to describe associations between milk immune characteristics and Cox proportional hazards (CPH) models to describe associations between milk immune characteristics and the instantaneous hazard of ID among infants. All milk immune characteristics were transformed by natural logarithm for CPH models due to substantial right skew. Because the modal value for both IL-6 response to stimuli variables was 1 (no change), we characterized these predictor variables two ways in CPH models: first, as a binary variable for any/no increase in IL-6 (IL-6 response > 1.0 vs ≤ 1.0), and second, as the *ln*-transformed continuous variable. For ease of interpretation of continuous, *ln*-transformed predictor variables, we present both hazard ratios (HR; exp(β)) and the impact of two-fold increase in the magnitude of a cytokine response (exp(β*ln(2))−1). Infant characteristics were included as control variables in CPH models if they were independently associated with any ID outcome or if their inclusion appreciably altered the estimated HR for a predictor of interest (milk immune characteristics). Interactions between infant and milk immune characteristics were evaluated to assess whether associations of interest differed by infant characteristics. Proportionality of hazards for all ‘full’ models was tested using the Schoenfeld test; LMAX measures of influence were used to assess the presence of particularly influential data points. All models were estimated in Stata SE 17.0 software.

## RESULTS

Complete maternal and infant birthdate information and adequate transitional/mature milk specimen for assessment of baseline milk content and cytokine responses to *S. enterica* were provided by 96 mothers; adequate specimen for additional evaluation of cytokine responses to *E. coli* was provided by 85 of these mothers (Supplementary Table S1).

Secretory IgA, baseline IL-6, and IL-6 responses to *S. enterica* and *E. coli* varied widely (Table 1, Supplementary Figure S1). The geometric mean IL-6 response to *S. enterica* was 4.2 and the geometric mean IL-6 response to *E. coli* was 3.8. By contrast, our assay captured little variation in IL-10 or IFN-γ in baseline or stimulated specimens: after incubation with *S. enterica* and *E. coli*, 94.8% and 100% of specimens, respectively, exhibited no change in IL-10, and 96.9% and 97.7% exhibited no change in IFN-γ. Thus, we focused in subsequent analyses on sIgA and IL-6; because a portion of IL-6 responses could be characterized as ‘no increase’ (universal low IL-6 both before and after incubation), we analyze these as both binary (increase/no increase) and continuous variables.

**Table 1.** Milk immune characteristics

	*N*	Geometric mean	Range	
Baseline^a^				
Secretory immunoglobulin A (sIgA; µg/ml)	96	931.2	320.8	6001.4
Interleukin-6 (pg/ml)	96	11.4	8.6	373.8
*Salmonella enterica*				
*S. enterica*-stimulated interleukin-6 (pg/ml)	96	47.7	8.6	2156.1
*S. enterica*-stimulated interleukin-10 (pg/ml)	96	37.2	35.3	2446.5
*S. enterica*-stimulated interferon-γ (pg/ml)	96	6.7	6.7	12.2
Interleukin-6 response^b^ to *S. enterica*	96	4.2	0.04	87.9
Interleukin-10 response to *S. enterica*	96	1.1	1.0	69.3
Interferon-γ response to *S. enterica*	96	1.0	0.7	1.8
*Escherichia coli*				
*E. coli*-stimulated interleukin-6 (pg/ml)	85	43.6	8.6	1009.5
*E. coli*-stimulated interferon-γ (pg/ml)	85	6.9	6.7	35.9
Interleukin-6 response to *E. coli*	85	3.8	0.1	104.9
Interferon-γ response to *E. coli*	85	1.0	1.0	5.1

a Little variation in IL-10 or IFN-γ was observed in baseline specimens. The universal low IL-6 was assigned to 76 baseline specimens, 18 *S. enterica-*stimulated specimens and 19 *E. coli*-stimulated specimens; the universal low IL-10 was assigned to all baseline and *E. coli*-stimulated specimens and 91 *S. enterica-*stimulated specimens; the universal low IFN-γ was assigned to 95 baseline specimens, 95 *S. enterica*-stimulated specimens and 83 *E. coli-*stimulated specimens.

b [*S. enterica* or *E. coli-*stimulated cytokine concentration]/[Baseline cytokine concentration].

When characterized as a binary variable, IL-6 responses to *S. enterica* were more common (81.3%) than IL-6 responses to *E. coli* (77.7%). Baseline IL-6, IL-6 responses, and sIgA were generally positively associated (Supplementary Table S2). In particular, there was a strong correlation between IL-6 responses to *S. enterica* and *E. coli* (Spearman’s rho: 0.60; *P*: 0.000; Supplementary Figure S2).

Infectious diseases were common among participating infants (Table 2), particularly respiratory infections (∼2 cases per 100 children per day). In bivariate CPH models, sIgA and milk *in vitro* IL-6 response to *S. enterica* (as either a binary or continuous variable) were inversely associated with respiratory ID (Supplementary Table S3a). The IL-6 response to *E. coli,* when characterized as a binary variable, was inversely associated with respiratory ID; when characterized as a continuous variable, the IL-6 response to *E. coli* was positively associated with gastrointestinal ID. In a multivariate model of milk immune characteristics (Supplementary Table S3b), milk *in vitro* IL-6 responses to *S. enterica* and *E. coli* stood out as predictors of infant ID risk.

**Table 2.** Incidence of infectious diseases among infants (cases per 100 child-days)

	Cases^a^	Incidence (males)	Incidence (females)	Incidence (total)
Any respiratory infection	60	2.14	1.90	2.02
Upper respiratory tract infection	40	1.26	0.87	1.06
Pneumonia	35	0.67	1.07	0.85
Gastrointestinal infection	30	0.86	0.56	0.71
Any infectious disease^b^	72	3.14	2.52	2.82

a Includes the first diagnosis within each category for each child; some children were cases within multiple categories at the same time point, some were cases within multiple categories at different time points.

^b^ Other infectious diseases included skin, eye, and urinary tract infections; no episodes of malaria were identified.

**Figure 1.**
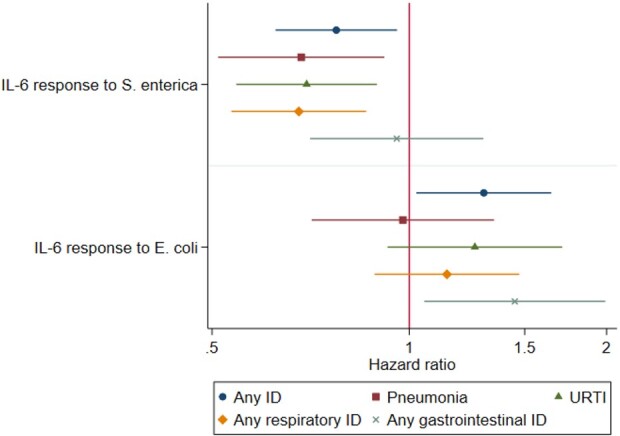
Estimated hazard ratios (HR) for milk immune activity from multivariate Cox proportional hazards models for each infectious disease outcome. Shapes represent the point estimate and bars represent the 95% confidence interval for each HR. Predictor variables are the ln-transformed continuous IL-6 response to stimulus (see Table 3). IL-6, interleukin-6; ID, infectious disease; URTI, upper respiratory tract infection

We assessed milk *in vitro* IL-6 responses to *S. enterica* and *E. coli* both separately and together in multivariate CPH models including infant age, sex, and size (length-for-age Z-score [LAZ]). The magnitude and direction of the effects of IL-6 responses to *S. enterica* changed little when evaluated separately or together with IL-6 responses to *E. coli*, suggesting the effect of IL-6 responses to *S. enterica* on infant ID risk was largely independent of the effect of IL-6 responses to *E. coli* (Supplementary Tables S4–S8). By contrast, IL-6 responses to *E. coli* were not independent of IL-6 responses to *S. enterica—*some associations were attenuated in magnitude or changed direction with inclusion of IL-6 response to *S. enterica*. We thus focused subsequent interpretation on the models including both responses (Table 3 and Figure 1). For all of these models, the assumption of proportionality of hazards was met and we identified no unduly influential data points.

**Table 3.** IL-6 responses to stimuli and infant characteristics as predictors of infectious disease in Cox proportional hazards models (*N* = 84)

	Respiratory infection (any)		Pneumonia		URTI		Gastrointestinal infection		Any infectious disease	
	HR	*P*	HR	*P*	HR	*P*	HR	*P*	HR	*P*
Binary IL-6 responses^a^										
IL-6 response to *S. enterica*	0.41	0.030	0.38	0.072	0.76	0.590	0.52	0.245	0.37	0.008
IL-6 response to *E. coli*	2.18	0.251	0.80	0.651	0.54	0.202	1.47	0.508	2.48	0.157
Infant age (months)	1.09	0.149	1.07	0.236	0.86	0.005	0.98	0.771	1.09	0.154
Male sex	1.24	0.491	0.46	0.060	1.65	0.161	1.41	0.403	1.32	0.326
Length-for-age Z-score^b^	0.91	0.266	1.25	0.100	0.59	0.007	1.00	0.991	0.99	0.899
Infant age*IL-6 resp to *E. coli*	0.83	0.021							0.85	0.042
LAZ*IL-6 resp to *S. enterica*					1.72	0.026				
Continuous IL-6 responses^c^										
IL-6 response to *S. enterica*	0.68	0.001	0.68	0.011	0.70	0.004	0.96	0.776	0.77	0.019
IL-6 response to *E. coli*	1.14	0.308	0.98	0.890	1.26	0.142	1.45	0.022	1.30	0.030
Infant age (months)	0.93	0.084	1.03	0.596	0.88	0.008	0.98	0.772	0.96	0.265
Male sex	1.05	0.864	0.51	0.113	1.69	0.155	1.43	0.383	1.14	0.628
Length-for-age Z-score^b^	0.95	0.564	1.27	0.088	0.91	0.308	0.98	0.856	1.00	0.959

a Any increase in IL-6 after incubation with stimulus (i.e. IL-6 response > 1).

b WHO Child Growth Standards (2006) [38].

c *ln*-transformed IL-6 response to stimulus; URTI, upper respiratory tract infection.

When characterized as binary variables (increase/no increase), the IL-6 response to *S. enterica* was associated with lower risk for ID, while the IL-6 response to *E. coli* was generally unassociated with ID risk. When characterized as continuous variables, IL-6 responses to *S. enterica* and *E. coli* were generally opposite in effect, with the IL-6 response to *S. enterica* inversely associated with respiratory ID (for any respiratory ID: HR: 0.68; 95% confidence interval [CI]: 0.54, 0.86; *P*: 0.001; for pneumonia: HR: 0.68; 95% CI: 0.51, 0.92; *P*: 0.011; for URTI: HR: 0.70; 95% CI: 0.54, 0.89; *P*: 0.004) and the IL-6 response to *E. coli* positively associated with gastrointestinal ID (HR: 1.44; 95% CI: 1.05, 1.99; *P*: 0.022). Each doubling of the IL-6 response to *S. enterica* was associated with 23% lower risk for any respiratory infection; by contrast, each doubling of the IL-6 response to *E. coli* was associated with a 29% increase in risk for gastrointestinal infection. This produced opposite effects on risk for ID overall: each doubling of the IL-6 response to *S. enterica* was associated with 17% lower risk for any ID (HR: 0.77; 95% CI: 0.62, 0.96; *P*: 0.019), while each doubling of the IL-6 response to *E. coli* was associated with 20% higher risk for any ID (HR: 1.30; 95% CI: 1.03, 1.65; *P*: 0.030).

Pneumonia risk was lower among male infants. URTI risk decreased with advancing infant age. LAZ was positively associated with pneumonia risk and inversely associated with URTI risk, depending on how milk immune responses were modeled. Infant characteristics that were neither confounders of the effect of milk IL-6 responses, nor independent predictors of infant ID risk, included preterm birth, number of siblings and weight-for-length Z-score. Low birthweight was positively associated with ID risk in some models, but was not included in final models because it was uncommon (five infants) and did not confound the effects of IL-6 responses.

Assessment of interactions suggests that associations between milk immune activity and ID outcomes may vary with infant characteristics, but only when IL-6 responses were modeled as binary (increase/no increase). Interaction between IL-6 response to *E. coli* and infant age suggests that IL-6 response to *E. coli*’s associations with respiratory infections and any ID diminished with advancing infant age*.* Similarly, the association between IL-6 response to *S. enterica* and URTI differed by infants’ LAZ. Overall, models do not suggest major differences in the effects of milk immune activity by infant characteristics.

## DISCUSSION

Affording protection against infection while minimizing harm due to inappropriate activity is one of the central evolutionary challenges faced by the immune system. Our findings suggest that this fundamental tension plays out in the ISOM as a tradeoff between appropriately directed proinflammatory activity and proinflammatory activity directed at benign targets.

We interpret the milk *in vitro* IL-6 response to *S. enterica* as a biomarker of the ISOM’s capacity for appropriate proinflammatory activity: *S. enterica* causes disease and is not part of the normal intestinal flora, and the IL-6 response to *S. enterica* was associated with lower risk for respiratory ID among infants. Our results suggest that milk exhibiting the capacity for appropriately directed proinflammatory immune activity (per the IL-6 response to *S. enterica*) can dramatically lower infants’ risk for ID. This is consistent with our expectation that ISOM creates a mother–infant immune axis that protects infants against ID.

The observed protective effect was limited to respiratory infections; proinflammatory immune responses to *S. enterica* were **not** associated with lower risk for gastrointestinal infections, even though we might have expected the ISOM to have its strongest protective effect in the gastrointestinal tract, via direct, *in situ* action against infectious agents. Our findings are more consistent with proinflammatory milk immune activity acting via support of systemic immune responses in infants than via direct action in the gut against invading infectious agents. This is consistent with research in animal models demonstrating that ISOM factors, particularly lymphocytes, move through gut-associated lymphoid tissue to infant immune tissues (e.g. thymus, spleen) to act systemically, in conjunction with the offspring’s immune system, to decrease ID risk [10, 11]. Why appropriate proinflammatory activity was not also associated with lower risk for gastrointestinal ID is unclear.

Because *E. coli* is a normal constituent of the human gut, it can be considered a relatively benign and potentially inappropriate target for milk proinflammatory immune activity, and milk *in vitro* IL-6 response to it a biomarker of the ISOM’s capacity for misdirected proinflammatory activity. Our results suggest that such activity may increase risk for gastrointestinal infections only at higher levels (i.e. a positive association with gastrointestinal ID was apparent for the continuous IL-6 response to *E. coli* variable, but not the binary variable that captured any versus no IL-6 increase in response to *E. coli*). Multiple pathways may be at work in this increased risk; however, because we observed it only for gastrointestinal infections, we suspect that the effect is primarily attributable to activity of the ISOM in the infant gut. Milk immune activity targeting *E. coli* and other relatively benign organisms may disrupt the gut microbiota in ways that create opportunities for infectious agents [40–45].

Independent effects of infant age (decreasing URTI risk) and LAZ (increasing risk for pneumonia) on ID risk were likely mediated by infants’ own immune systems, which generally grow in competence as infants mature [46] and may be compromised during periods of rapid infant growth. There is growing evidence that growth is compromised in favor of immunity where ID risk is high [47, 48]; the converse—compromised immunity during or after growth—may also be at work [49].

That milk proinflammatory activity in the presence of *S. enterica* (an appropriate target) and *E. coli* (a benign target) have divergent effects on ID risk but were positively correlated is consistent with known tradeoffs in immunity: the capacity for protective immune activity often comes at the inherent peril of misdirected immune activity and collateral damage, including immune responses that can do more harm than good [18, 25]. In this case, our findings point to a tradeoff in the mother–infant immune axis between the protection afforded by ISOM activity (manifest in our study as lower risk for respiratory ID) and the ISOM’s capacity for misdirected activity (manifest in our study as elevated risk for gastrointestinal ID). An evolutionary perspective suggests that, in the context of this tradeoff, ISOM activity presents an optimization problem to mothers and mother–infant dyads. Multiple factors are likely to influence optimal ISOM activity, including the local incidence and severity of ID, prevalent disease-causing organisms, and other characteristics of the dyad’s environment, as well as maternal and infant characteristics [17, 30, 50].

Evolutionary perspectives on the ISOM [16, 17, 30, 50] remain sparse, but stand to provide a great deal of insight into human evolutionary biology and infant health. Our findings make clear that, as elsewhere in the immune system, ISOM proinflammatory activity has both benefits and costs, and caution against simple interpretations of milk immune content or activity as exclusively beneficial to infants. Indeed, the ISOM may be a particularly informative focus for research on the tradeoffs inherent in immune defense. Infancy is a time of high vulnerability to ID and high risk for mortality, contributing to the strong force of natural selection across early childhood [51]. Our findings suggest an important new area of research: whether and how proinflammatory milk immune activity is optimized, considering the multifactorial effects of ISOM activity and tradeoffs inherent therein. It will be important as this body of literature grows for research to assess variation in milk immune activity across settings and ID ecologies. It will also be important to assess tradeoffs more broadly by investigating the longer-term effects of milk immune activity on children’s risk for ID, as well as immune-mediated diseases, such as allergy and autoimmunity, and health and fitness outcomes, such as growth.

### Limitations

Our study design may have missed ID episodes (e.g. those that caused no symptoms, mild symptoms or symptoms that participants perceived to be unproblematic). Our sample size and follow-up period were inadequate to describe risk for uncommon ID outcomes, which may constitute important threats to infants.

We may have under-characterized *in vitro* responses if cytokines increased but remained below the assay limit of detection both before and after incubation. We relied on calibrators to calculate inter-assay variability in the absence of assay controls. It is possible that a different assay or assays would overcome these limitations.

In addition, although milk immune activity measured *in vitro* provides an informative, system-level outcome to describe the ISOM, the *in vitro* environment does not generalize to the infant gut. We were able to describe proinflammatory immune activity *in vitro*, but can conclude little about other aspects of ISOM regulation, including anti-inflammatory activity (IL-10 responses) or type I activity (IFN-γ responses). Such regulation may occur *in vivo* (e.g. in the presence of infant gut-associated lymphoid tissue) in ways that are not replicable *in vitro*.

Finally, our ability to test causal hypotheses is limited. Our observations support the hypothesis that milk immune activity directed at pathogenic targets protects against ID, while milk immune activity directed at benign targets increases risk for ID. However, phenotypic correlation—e.g. generally healthier mothers having coincidentally healthier infants and correlated milk immune activity—can only definitively be excluded with an experimental design that allocates infants to receive milk of varying immune activity from unrelated mothers.

### Conclusions

Human milk’s capacity for immune activity has complex, multifactorial effects, including both benefits and costs to infants in terms of their ID risk: the capacity for appropriate proinflammatory activity may protect against respiratory infection, including pneumonia, while the capacity for misdirected proinflammatory activity may increase risk for gastrointestinal infection. Although opposite in their effects on infant ID risk, appropriate and misdirected milk immune activity were positively correlated, demonstrating a tradeoff in the ISOM and raising important questions about how mother–infant dyads manage this tradeoff, and with what consequences for infant health.

## Supplementary Material

eoac020_Supplementary_Data

## References

[1] Abdel-Hafeez EH , Belal US, Abdellatif MZM. Breast-feeding protects infantile diarrhea caused by intestinal protozoan infections. Korean J Parasitol 2013;51:519–24. 24327776 10.3347/kjp.2013.51.5.519 PMC3857498

[2] Hanson LA. Breastfeeding provides passive and likely long-lasting active immunity. Ann Allergy Asthma Immunol 1998;81:523–37. 9892025 10.1016/S1081-1206(10)62704-4

[3] Oddy WH. Breastfeeding protects against illness and infection in infants and children: a review of the evidence. Breastfeed Rev 2001;9:11–9. 11550600

[4] Quigley MA , Kelly YJ, Sacker A. Breastfeeding and hospitalization for diarrheal and respiratory infection in the United Kingdom Millennium Cohort Study. Pediatrics 2007;119:e837–42. 17403827 10.1542/peds.2006-2256

[5] Andreas NJ , Kampmann B, Mehring Le-Doare K. Human breast milk: review on its composition and bioactivity. Early Hum Dev 2015;91:629–36. 26375355 10.1016/j.earlhumdev.2015.08.013

[6] Field CJ. The immunological components of human milk and their effect on immune development in infants. J Nutr 2005;135:1–4. 15623823 10.1093/jn/135.1.1

[7] Goldman AS. The immune system of human milk: antimicrobial, antiinflammatory and immunomodulating properties. Pediatr Infect Dis J 1993;12:664–73. 8414780 10.1097/00006454-199308000-00008

[8] Goldman AS. The immune system in human milk and the developing infant. Breastfeed Med 2007;2:195–205. 18081456 10.1089/bfm.2007.0024

[9] Newburg DS. Innate immunity and human milk. J Nutr 2005;135:1308–12. 15867330 10.1093/jn/135.5.1308

[10] Cabinian A , Sinsimer D, Tang M et al Transfer of maternal immune cells by breastfeeding: maternal cytotoxic T lymphocytes present in breast milk localize in the Peyer’s patches of the nursed infant. PLoS One 2016;11:e0156762. 27285085 10.1371/journal.pone.0156762 PMC4902239

[11] Ghosh MK , Nguyen V, Muller HK, Walker AM. Maternal milk T cells drive development of transgenerational Th1 immunity in offspring thymus. J Immunol 2016;197:2290–6. 27496970 10.4049/jimmunol.1502483 PMC5009876

[12] Ruiz L , Espinosa-Martos I, García-Carral C et al What’s normal? Immune profiling of human milk from healthy women living in different geographical and socioeconomic settings. Front Immunol 2017;8:696. 28713365 10.3389/fimmu.2017.00696 PMC5492702

[13] Hayani KC , Guerrero ML, Morrow AL et al Concentration of milk secretory immunoglobulin A against *Shigella* virulence plasmid-associated antigens as a predictor of symptom status in *Shigella*-infected breast-fed infants. J Pediatr 1992;121:852–6. 1447644 10.1016/s0022-3476(05)80327-0

[14] Walterspiel JN , Morrow AL, Pickering LK et al Secretory anti-*Giardia lamblia* antibodies in human milk: protective effect against diarrhea. Pediatrics 1994;93:28–31. 8265319

[15] Ruiz-Palacios GM , Calva JJ, Pickering LK et al Protection of breast-fed infants against *Campylobacter* diarrhea by antibodies in human milk. J Pediatr 1990;116:707–13. 2329419 10.1016/s0022-3476(05)82652-6

[16] Breakey AA , Hinde K, Valeggia CR et al Illness in breastfeeding infants relates to concentration of lactoferrin and secretory immunoglobulin A in mother’s milk. Evol Med Public Health 2015;2015:21–32. 25608691 10.1093/emph/eov002 PMC4334701

[17] Miller EM. Ecological immunity of human milk: life history perspectives from the United States and Kenya. Am J Phy Anthropol 2018;167:389–99. 10.1002/ajpa.23639 30129152

[18] Silverstein AM , Rose NR. There is only one immune system! The view from immunopathology. Semin Immunol 2000;12:173–8. 10910736 10.1006/smim.2000.0228

[19] Newton AH , Cardani A, Braciale TJ. The host immune response in respiratory virus infection: balancing virus clearance and immunopathology. Semin Immunopathol 2016;38:471–82. 26965109 10.1007/s00281-016-0558-0 PMC4896975

[20] Silver KL , Higgins SJ, McDonald CR, Kain KC. Complement driven innate immune response to malaria: fueling severe malarial diseases. Cell Microbiol 2010;12:1036–45. 20545944 10.1111/j.1462-5822.2010.01492.x

[21] Singla M , Kar M, Sethi T et al Immune response to dengue virus infection in pediatric patients in New Delhi, India—association of viremia, inflammatory mediators and monocytes with disease severity. PLoS Negl Trop Dis 2016;10:e0004497. 26982706 10.1371/journal.pntd.0004497 PMC4794248

[22] Daley D. The evolution of the hygiene hypothesis: the role of early-life exposures to viruses and microbes and their relationship to asthma and allergic diseases. Curr Opin Allergy Clin Immunol 2014;14:390–6. 25102107 10.1097/ACI.0000000000000101

[23] Sartor RB. Role of commensal enteric bacteria in the pathogenesis of immune-mediated intestinal inflammation: lessons from animal models and implications for translational research. J Pediatr Gastroenterol Nutr 2005;40:S30. 15805841 10.1097/00005176-200504001-00018

[24] Ruff WE , Greiling TM, Kriegel MA. Host–microbiota interactions in immune-mediated diseases. Nature Rev Microbiol 2020;18:521–38. 32457482 10.1038/s41579-020-0367-2

[25] Bennett JM , Reeves G, Billman GE, Sturmberg JP. Inflammation–nature’s way to efficiently respond to all types of challenges: implications for understanding and managing “the epidemic” of chronic diseases. Front Med 2018; 5:316. 10.3389/fmed.2018.00316 PMC6277637 30538987

[26] Muehlenbein MP , Hirschtick JL, Bonner JZ, Swartz AM. Toward quantifying the usage costs of human immunity: altered metabolic rates and hormone levels during acute immune activation in men. Am J Hum Biol 2010;22:546–57. 20309883 10.1002/ajhb.21045

[27] McDade TW. Life history theory and the immune system: steps toward a human ecological immunology. Am J Phys Anthropol 2003;122:100–25. 10.1002/ajpa.10398 14666535

[28] McDade TW. The ecologies of human immune function. Annu Rev Anthropol 2005;34:495–521.

[29] Long KZ , Nanthakumar N. Energetic and nutritional regulation of the adaptive immune response and trade-offs in ecological immunology. Am J Hum Biol 2004;16:499–507. 15368598 10.1002/ajhb.20064

[30] Fujita M , Wander K, Paredes Ruvalcaba N, Brindle E. Human milk sIgA antibody in relation to maternal nutrition and infant vulnerability in northern Kenya. Evol Med Public Health 2019;2019:201–11. 32405414 10.1093/emph/eoz030 PMC7216193

[31] Nishimoto N , Kishimoto T. Interleukin 6: from bench to bedside. Nat Clin Pract Rheumatol 2006;2:619–26. 17075601 10.1038/ncprheum0338

[32] Rincon M. Interleukin-6: from an inflammatory marker to a target for inflammatory diseases. Trends Immunol 2012;33:571–7. 22883707 10.1016/j.it.2012.07.003

[33] Mosser DM , Zhang X. Interleukin-10: new perspectives on an old cytokine. Immunol Rev 2008;226:205–18. 19161426 10.1111/j.1600-065X.2008.00706.x PMC2724982

[34] Sabat R , Grütz G, Warszawska K et al Biology of interleukin-10. Cytokine Growth Factor Rev 2010;21:331–44. 21115385 10.1016/j.cytogfr.2010.09.002

[35] Annunziato F , Cosmi L, Liotta F et al Human T helper type 1 dichotomy: origin, phenotype and biological activities. Immunology 2015;144:343–51. 10.1111/imm.12399 PMC4557671 25284714

[36] Wander K , Fujita M, Spathis R et al In vitro stimulation of whole milk specimens: A field-friendly method to assess milk immune activity. J Hum Lact 2021;37:736–45. 33788640 10.1177/0890334421999628

[37] Ministry of Health, Community Development, Gender, Elderly and Children (MoHCDGEC) [Tanzania Mainland], Ministry of Health (MoH) [Zanzibar], National Bureau of Statistics (NBS), Office of the Chief Government Statistician (OCGS), and ICF. *Tanzania Demographic and Health Survey and Malaria Indicator Survey (TDHS-MIS) 2015-16*. Dar es Salaam, Tanzania, and Rockville, Maryland, USA: MoHCDGEC, MoH, NBS, OCGS, and ICF, 2016.

[38] Hassiotou F , Hepworth AR, Metzger P et al Maternal and infant infections stimulate a rapid leukocyte response in breastmilk. Clin Transl Immunol 2013;2:e3. 10.1038/cti.2013.1 PMC4232055 25505951

[39] Leroy J. 2011 *ZSCORE06: Stata module to calculate anthropometric z-scores using the 2006 WHO child growth standards.* Statistical Software Components S457279. Boston College Department of Economics. https://ideas.repec.org/c/boc/bocode/s457279.html.

[40] Ghosh S , Padalia J, Moonah S. Tissue destruction caused by *Entamoeba histolytica* parasite: Cell death, inflammation, invasion, and the gut microbiome. Curr Clin Micro Rpt 2019;6:51–7. 10.1007/s40588-019-0113-6 PMC6449278 31008019

[41] Leon-Coria A , Kumar M, Moreau F, Chadee K. Defining cooperative roles for colonic microbiota and Muc2 mucin in mediating innate host defense against *Entamoeba histolytica*. PLoS Pathog 2018;14:e1007466. 30500860 10.1371/journal.ppat.1007466 PMC6268003

[42] Stensvold CR , van der Giezen M. Associations between gut microbiota and common luminal intestinal parasites. Trends Parasitol 2018;34:369–77. 29567298 10.1016/j.pt.2018.02.004

[43] Croswell A , Amir E, Teggatz P et al Prolonged impact of antibiotics on intestinal microbial ecology and susceptibility to enteric *Salmonella* infection. Infect Immun 2009;77:2741–53. 19380465 10.1128/IAI.00006-09 PMC2708550

[44] Jacobson A , Lam L, Rajendram M et al A gut commensal-produced metabolite mediates colonization resistance to *Salmonella* infection. Cell Host Microbe 2018;24:296–307.e7. 30057174 10.1016/j.chom.2018.07.002 PMC6223613

[45] Kaiser BLD et al A multi-omic view of host-pathogen-commensal interplay in *Salmonella*-mediated intestinal infection. PLoS One 2013;8:e67155. 23840608 10.1371/journal.pone.0067155 PMC3694140

[46] Holt PG. Postnatal maturation of immune competence during infancy and childhood. Pediatr Allergy Immunol 1995;6:59–70. 7581722 10.1111/j.1399-3038.1995.tb00261.x

[47] McDade TW , Reyes‐García V, Tanner S, Huanca T et al Maintenance versus growth: investigating the costs of immune activation among children in lowland Bolivia. Am J Phys Anthropol 2008;136:478–85. 18383156 10.1002/ajpa.20831

[48] Garcia AR , Blackwell AD, Trumble BC et al Evidence for height and immune function tradeoffs among preadolescents in a high pathogen population. Evol Med Public Health 2020;2020:86–99. 32983534 10.1093/emph/eoaa017 PMC7502263

[49] Wander K , Shell-Duncan B, Brindle E, O'Connor K. Predictors of delayed‐type hypersensitivity to *Candida albicans* and anti‐Epstein‐Barr virus antibody among children in Kilimanjaro, Tanzania. Am J Phys Anthropol 2013;151:183–91. 23460387 10.1002/ajpa.22250 PMC4005610

[50] Klein LD , Huang J, Quinn EA et al Variation among populations in the immune protein composition of mother’s milk reflects subsistence pattern. Evol Med Public Health 2018;2018:230–45. 30430010 10.1093/emph/eoy031 PMC6222208

[51] Jones JH. The force of selection on the human life cycle. Evol Hum Behav 2009;30:305–14. 22003281 10.1016/j.evolhumbehav.2009.01.005 PMC3193054

