# Supplementary material for: Tradeoffs in milk immunity affect infant infectious disease risk
Source: Evol Med Public Health. 2022 Jun 13;10(1):295–304. doi: 10.1093/emph/eoac020 (PMC9233416; doi:10.1093/emph/eoac020)
Supplement: eoac020_Supplementary_Data [file eoac020_supplementary_data.pdf]

Tradeoffs in milk immunity affect infant infectious disease risk

Supplemental Information

Katherine Wander, Masako Fujita, Siobhan M Mattison, Margaret Duris, Megan Gauck, Tessa Hopt, Katherine Lacy, Angela Foligno, Rebecca Ulloa, Connor Dodge, Frida Mowo, Ireen Kiwelu, Blandina T Mmbaga

Katherine Wander

Email: katherinewander@binghamton.edu

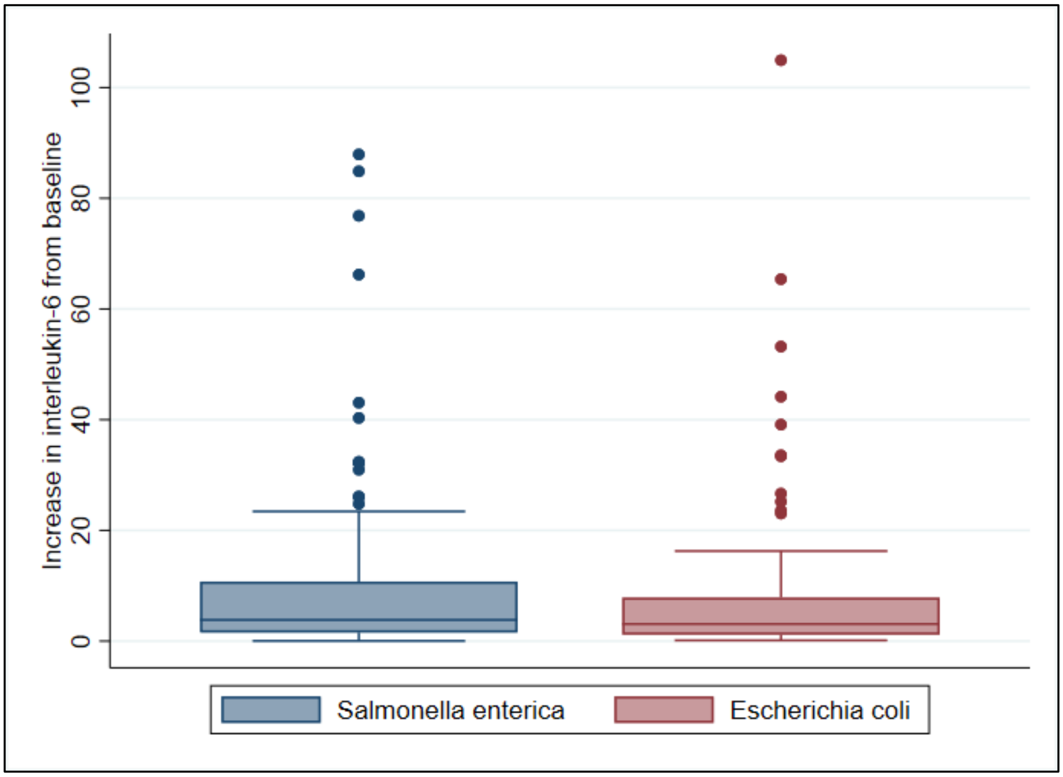

Figure S1. Box plots of the *in vitro* interleukin-6 response to *Salmonella enterica* (left) and *Escherichia coli* (right). Substantial right skew was apparent in both variables.

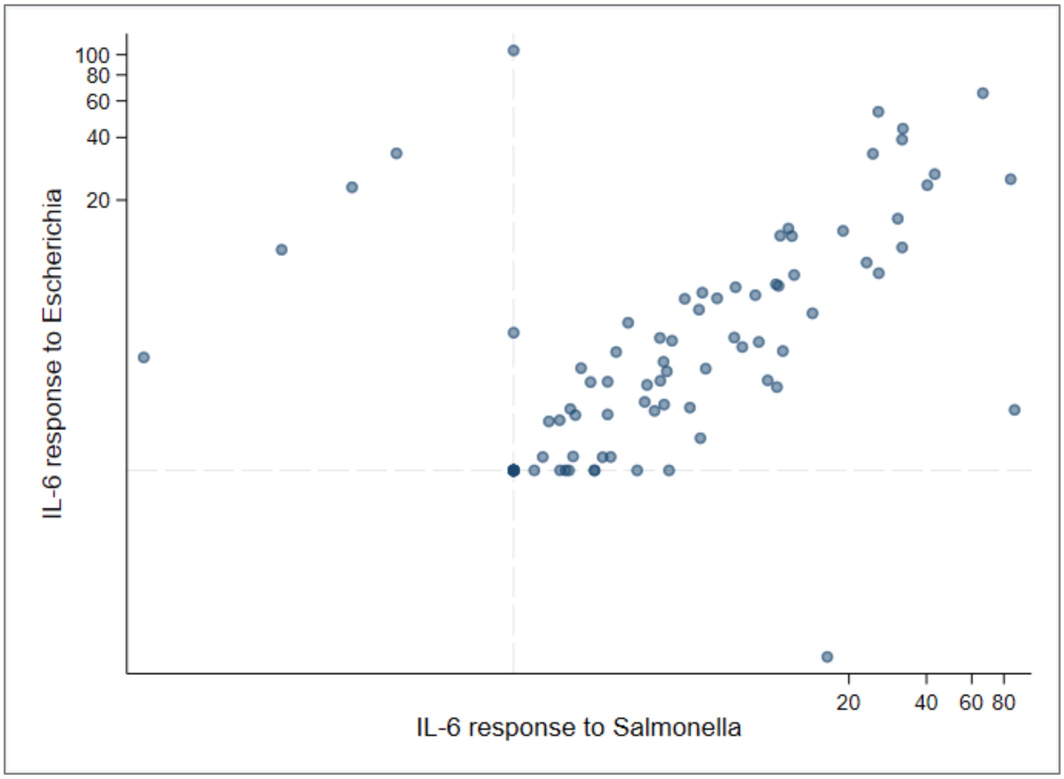

Figure S2. *In vitro* interleukin (IL)-6 responses to two bacterial agents, *Escherichia coli* and *Salmonella enterica*. Reference lines show IL-6 response of 1, or no increase in IL-6 after incubation with bacterial stimulant.

**Table S1. Sample Characteristics**				
**Mothers**	**N responding**	**Mean**	**Range**	
Age (years)	96	28.3	18.9	44.5
Total pregnancies	96	2.8	1	10
Total live births	96	2.5	1	6
Height (cm)	96	160.4	143.5	173.5
Weight (kg)	96	66.4	41.5	93.7
BMI	96	25.7	16.6	36.6
	**N responding**	**N**		**%**
Education	96			
None		1		1.0
Primary (some)		3		3.1
Primary (completed)		49		51.0
Secondary (some)		18		18.8
Secondary (completed Form 4)		21		21.9
Secondary (completed Form 6 or more)		4		4.2
Marital status	93			
Unmarried		12		12.9
Widowed		1		1.1
Co-habiting		8		8.6
Divorced		1		1.1
Married		71		76.3
Recent (preceding two weeks) health:				
Fever/chills	96	14		14.6
Aches	96	8		8.3
Painful urination	96	8		8.3
Cough	96	33		34.4
Sneeze	96	15		15.6
Sore throat	96	1		1.0
Sinus pain	96	2		2.1
Earache	96	3		3.1
Rash (skin)	96	1		1.0
Diarrhea	96	2		2.1
Stomachache	96	8		8.3
Currently pregnant	94	4		4.3
Resumed menstruation	96	66		73.3
Anemia (hemoglobin < 12 g/dl)	95	33		34.7
Overweight or obese (BMI > 25)	96	49		51.0
Obese (BMI >30)	96	20		20.8
**Infants**	**N responding**	**Mean**	**Range**	
Age (months)	96	7.1	0.4	13.6
Weight at birth (kg)*	95	3.2	1.0	5.0
Length for age Z-score†	95	0.1	-4.9	3.3
Weight for length Z-score†	95	-0.1	-3.9	3.2
	**N responding**	**N**		**%**
Sex	95			
Female		48		50.5
Male		47		49.5
Low birthweight (<2.5 kg)	96	5		5.2
Recent symptoms	96			
Fever/chills		35		36.5
Weakness		4		4.2
Eye discharge/swelling		15		15.6

Cough		54	56.3
Sneeze		57	59.4
Congestion		11	11.5
Earache		6	6.3
Rash (skin)		12	12.5
Diarrhea		6	6.3
Current supplementation:			
Water	95	76	80.0
Cow’s milk	95	70	73.7
Porridge (“uji”)	94	66	70.2
Other supplemental food	93	60	64.5
Family foods	95	52	55.9
**Pregnancy/birth**	**N responding**	**N**	**%**
Mode of birth	92		
Vaginal		72	78.3
Cesarean		20	21.7
Term	77		
Preterm (<38 wks)		18	23.4
Term (38-42 wks)		52	67.5
Postterm (>42 wks)		7	9.1
Birth complications	96	3	3.1
Congenital abnormalities	96	1	1.0
Infant received supplement (in hospital):	96		
Water		8	8.3
Formula		2	2.1
Other supplement		2	2.1
**Household**	**N responding**	**N**	**%**
Dwelling material	95		
Wood/earth		11	11.6
Cement		84	88.4
Electricity	93		
Yes		68	73.1
No		25	26.9

*One implausibly high value (7.7kg) was excluded. †WHO Child Growth Standards (2006)
‡Excludes first-born infants

**Table S2. Associations between milk measures of immunity** (Spearman's rho; *p-value*)	sIgA	Baseline IL-6	IL-6 response to *S. enterica*
Baseline IL-6	0.37 *(0.0005)*	--	
IL-6 response to *S. enterica*	0.28 *(0.0104)*	0.06 *(0.5872)*	--
IL-6 response to *E. coli*	0.41 *(0.0001)*	0.28 *(0.0104)*	0.60 *(0.0000)*

Table S3. Milk Immunity Measures as Predictors of Infectious Disease Risk

	Respiratory infection (any)		Pneumonia		URTI		Gastrointestinal infection		Any ID	
a. Bivariate Models	HR	p	HR	p	HR	p	HR	p	HR	p
sIgA (μg/ml)*	**0.54**	**0.026**	0.55	0.121	0.61	0.148	0.93	0.850	0.75	0.221
Baseline IL-6 (pg/ml)*	0.94	0.755	0.73	0.356	1.11	0.590	1.36	0.139	0.93	0.666
IL-6 response to *S. enterica* (binary)	**0.36**	**0.001**	**0.46**	**0.041**	**0.43**	**0.019**	0.66	0.343	**0.39**	**0.001**
IL-6 response to *S. enterica**	**0.72**	**0.004**	**0.74**	**0.020**	**0.79**	**0.037**	1.18	0.244	0.86	0.148
IL-6 response to *E. coli* (binary)	**0.43**	**0.005**	0.56	0.155	0.51	0.061	0.96	0.938	0.60	0.072
IL-6 response to *E. coli**	0.92	0.502	0.88	0.403	1.00	0.988	**1.43**	**0.018**	1.13	0.267
b. Multivariate Model†										
sIgA (μg/ml)*0.	0.78	0.490	0.68	0.473	0.73	0.469	0.56	0.252	0.88	0.693
IL-6 response to *S. enterica**	**0.67**	**0.001**	**0.71**	**0.020**	**0.70**	**0.002**	0.96	0.804	**0.78**	**0.018**
IL-6 response to *E. coli**	1.21	0.213	1.04	0.821	1.35	0.091	**1.59**	**0.009**	**1.32**	**0.037**
Infant age (months)	0.94	0.106	1.00	0.958	0.90	0.029	1.00	0.931	0.97	0.345

**ln*-transformed; †Baseline IL-6 was included in the calculation of IL-6 responses variables and so is excluded here.

Table S4. Full CPH models: any respiratory infection

	HR	95% CI	p
a. Bivariate models:			
sIgA*	0.54	0.32, 0.93	0.026
Baseline IL-6*	0.94	0.64, 1.39	0.755
Binary IL-6 response to *S. enterica*	0.36	0.20, 0.65	0.001
Continuous IL-6 response to *S. enterica**	0.72	0.58, 0.90	0.004
Binary IL-6 response to *E. coli*	0.43	0.24, 0.77	0.005
Continuous IL-6 response to *E. coli**	0.92	0.72, 1.17	0.502
b. Multivariate model, immune content:			
sIgA*	0.78	0.38, 1.60	0.490
Continuous IL-6 response to *S. enterica**	0.67	0.53, 0.85	0.001
Continuous IL-6 response to *E. coli**	1.21	0.90, 1.62	0.213
Infant age (months)	0.94	0.87, 1.01	0.106
c. Multivariate model, binary IL-6 response to *S. enterica* (increase/no increase)			
IL-6 response to *S. enterica*	0.40	0.22, 0.74	0.004
Infant age (months)	0.94	0.88, 1.02	0.125
Male sex	1.23	0.71, 2.14	0.453
Length for age Z-score	0.93	0.80, 1.09	0.373
d. Multivariate model, continuous IL-6 response to *S. enterica*:			
IL-6 response to *S. enterica**	0.73	0.59, 0.91	0.005
Infant age (months)	0.92	0.86, 0.99	0.034
Male sex	1.22	0.71, 2.12	0.467
Length for age Z-score	0.95	0.81, 1.11	0.500
e. Multivariate model, binary IL-6 response to *E. coli* (increase/no increase)			
IL-6 response to *E. coli*	0.46	0.25, 0.85	0.014
Infante age (months)	0.97	0.90, 1.04	0.396
Male sex	1.01	0.56, 1.81	0.986
Length for age Z-score	0.92	0.79, 1.08	0.300
f. Multivariate model, IL-6 response to *E. coli*:			
IL-6 response to *E. coli**	0.93	0.73, 1.18	0.562
Infant age (months)	0.94	0.88, 1.02	0.148
Male sex	1.02	0.56, 1.85	0.944
Length-for-age Z-score	0.91	0.77, 1.07	0.250
g. Multivariate model, both binary IL-6 response to stimuli (increase/no increase)			
IL-6 response to *S. enterica*	0.41	0.18, 0.92	0.030
IL-6 response to *E. coli*	2.18	0.58, 8.24	0.251
Infant age (months)	1.09	0.97, 1.24	0.149
Male sex	1.24	0.68, 2.25	0.491
Length for age Z-score	0.91	0.78, 1.07	0.266
Infant age*IL-6 response to *E. coli*	0.83	0.70, 0.97	0.021
h. Multivariate model, both continuous IL-6 responses to stimuli:			
IL-6 response to *S. enterica**	0.68	0.54, 0.86	0.001
IL-6 response to *E. coli**	1.14	0.89, 1.47	0.308
Infant age (months)	0.93	0.86, 1.01	0.084
Male sex	1.05	0.58, 1.90	0.864
Length-for-age Z-score	0.95	0.81, 1.12	0.564

*ln-transformed

**Table S5. Full CPH models: pneumonia**			
	HR	95% CI	p
a. Bivariate models:			
sIgA*	0.55	0.26, 1.17	0.121
Baseline IL-6*	0.73	0.38, 1.42	0.356
Binary IL-6 response to *S. enterica*	0.46	0.22, 0.97	0.041
Continuous IL-6 response to *S. enterica**	0.74	0.58, 0.95	0.020
Binary IL-6 response to *E. coli*	0.56	0.26, 1.24	0.155
Continuous IL-6 response to *E. coli**	0.88	0.65, 1.19	0.403
b. Multivariate model, immune content:			
sIgA*	0.68	0.24, 1.93	0.473
Continuous IL-6 response to *S. enterica**	0.71	0.53, 0.95	0.020
Continuous IL-6 response to *E. coli**	1.04	0.73, 1.49	0.821
Infant age (months)	1.00	0.90, 1.11	0.958
c. Multivariate model, binary IL-6 response to *S. enterica* (increase/no increase):			
IL-6 response to *S. enterica*	0.34	0.15, 0.78	0.011
Infant age (months)	1.04	0.94, 1.16	0.348
Male sex	0.68	0.33, 1.37	0.280
Length for age Z-score	1.25	0.98, 1.59	0.071
d. Multivariate model, continuous IL-6 response to *S. enterica*:			
IL-6 response to *S. enterica**	0.71	0.54, 0.93	0.013
Infant age (months)	1.01	0.91, 1.11	0.898
Male sex	0.73	0.36, 1.49	0.393
Length for age Z-score	1.23	0.97, 1.58	0.090
e. Multivariate model, binary IL-6 response to *E. coli* (increase/no increase):			
IL-6 response to *E. coli*	0.51	0.23, 1.14	0.103
Infant age (months)	1.04	0.94, 1.16	0.458
Male sex	0.47	0.21, 1.06	0.069
Length for age Z-score	1.16	0.90, 1.48	0.251
f. Multivariate model, continuous IL-6 response to *E. coli*:			
IL-6 response to *E. coli**	0.89	0.65, 1.22	0.477
Infant age (months)	1.03	0.93, 1.15	0.565
Male sex	0.47	0.21, 1.08	0.075
Length for age Z-score	1.17	0.90, 1.52	0.244
g. Multivariate model, both binary IL-6 response to stimuli (increase/no increase):			
IL-6 response to *S. enterica*	0.38	0.13, 1.09	0.072
IL-6 response to *E. coli*	0.80	0.31, 2.09	0.651
Infant age (months)	1.07	0.96, 1.20	0.236
Male sex	0.46	0.20, 1.03	0.060
Length for age Z-score	1.25	0.96, 1.64	0.100
h. Multivariate model, both continuous IL-6 responses to stimuli:			
IL-6 response to *S. enterica**	0.68	0.51, 0.92	0.011
IL-6 response to *E. coli**	0.98	0.71, 1.35	0.890
Infant age (months)	1.03	0.92, 1.15	0.596
Male sex	0.51	0.23, 1.17	0.113
Length for age Z-score	1.27	0.97, 1.67	0.088

*ln-transformed

Table S6. Full CPH models: upper respiratory tract infections

	HR	95% CI	p
a. Bivariate models:			
sIgA*	0.61	0.32, 1.19	0.148
Baseline IL-6*	1.11	0.76, 1.63	0.590
Binary IL-6 response to *S. enterica*	0.43	0.22, 0.87	0.019
Continuous IL-6 response to *S. enterica**	0.79	0.63, 0.99	0.037
Binary IL-6 response to *E. coli*	0.51	0.25, 1.03	0.061
Continuous IL-6 response to *E. coli**	1.00	0.76, 1.32	0.988
b. Multivariate model, immune content:			
sIgA*	0.73	0.31, 1.70	0.469
IL-6 response to *S. enterica**	0.69	0.55, 0.87	0.002
IL-6 response to *E. coli**	1.35	0.95, 1.93	0.091
Infant age (months)	0.90	0.82, .099	0.029
c. Multivariate model, binary IL-6 response to *S. enterica* (increase/no increase)			
IL-6 response to *S. enterica*	0.57	0.26, 1.26	0.166
Infant age (months)	0.84	0.76, 0.93	0.001
Male sex	1.80	0.92, 3.53	0.089
Length for age Z-score	0.62	0.43, 0.88	0.007
LAZ*IL-6 response to *S. enterica*	1.56	1.02, 2.40	0.040
d. Multivariate model, continuous IL-6 response to *S. enterica*:			
IL-6 response to *S. enterica**	0.79	0.63, 1.00	0.047
Infant age (months)	0.87	0.80, 0.95	0.002
Male sex	1.70	0.86, 3.33	0.124
Length for age Z-score	0.88	0.73, 1.04	0.141
e. Multivariate model, binary IL-6 response to *E coli* (increase/no increase)			
IL-6 response to *E. coli*	0.61	0.29, 1.27	0.186
Infant age (months)	0.91	0.83, 1.00	0.047
Male sex	1.44	0.71, 2.91	0.317
Length for age Z-score	0.87	0.73, 1.04	0.115
f. Multivariate model, continuous IL-6 response to *E. coli*:			
IL-6 response to *E. coli**	1.00	0.76, 1.31	0.988
Infant age (months)	0.90	0.82, 0.98	0.018
Male sex	1.45	0.72, 2.93	0.296
Length for age Z-score	0.85	0.71, 1.03	0.097
g. Multivariate model, both binary IL-6 response to stimuli (increase/no increase)			
IL-6 response to *S. enterica*	0.76	0.28, 2.06	0.590
IL-6 response to *E. coli*	0.54	0.21, 1.39	0.202
Infant age (months)	0.86	0.78, 0.96	0.005
Male sex	1.65	0.82, 3.34	0.161
Length for age Z-score	0.59	0.41, 0.86	0.007
LAZ*IL-6 response to *S. enterica*	1.72	1.07, 2.77	0.026
d. Multivariate model, both continuous IL-6 responses to stimuli:			
IL-6 response to *S. enterica**	0.70	0.54, 0.89	0.004
IL-6 response to *E. coli**	1.26	0.93, 1.71	0.142
Infant age (months)	0.88	0.80, 0.97	0.008
Male sex	1.69	0.82, 3.47	0.155
Length for age Z-score	0.91	0.75, 1.09	0.308

**ln*-transformed

Table S7. Full CPH models: gastrointestinal infection

	HR	95% CI	p
a. Bivariate models:			
sIgA*	0.93	0.44, 1.95	0.850
Baseline IL-6*	1.36	0.90, 2.04	0.139
Binary IL-6 response to *S. enterica*	0.66	0.28, 1.55	0.343
Continuous IL-6 response to *S. enterica**	1.18	0.89, 1.56	0.244
Binary IL-6 response to *E. coli*	0.96	0.39, 2.39	0.938
Continuous IL-6 response to *E. coli**	1.43	1.06, 1.92	0.018
b. Multivariate immune content model:			
sIgA*	0.56	0.20, 1.51	0.252
Continuous IL-6 response to *S. enterica**	0.96	0.72, 1.29	0.804
IL-6 response to *E. coli**	1.58	1.12, 2.24	0.009
Infant age (months)	1.00	0.90, 1.13	0.931
c. Multivariate model, binary IL-6 response to *S. enterica* (increase/no increase)			
IL-6 response to *S. enterica*	0.73	0.30, 1.74	0.475
Infant age (months)	0.98	0.89, 1.08	0.676
Male sex	1.49	0.70, 3.19	0.304
Length for age Z-score	0.95	0.75, 1.21	0.677
d. Multivariate model, continuous IL-6 response to *S. enterica*			
IL-6 response to *S. enterica**	0.83	0.56, 1.23	0.357
Infant age (months)	0.98	0.88, 1.09	0.637
Male sex	0.53	0.17, 1.60	0.258
Length for age Z-score	0.87	0.68, 1.12	0.284
Sex*IL-6 response to *S. enterica*	1.92	1.14, 3.21	0.014
e. Multivariate model, binary IL-6 response to *E. coli* (increase/no increase)			
IL-6 response to *E. coli*	1.03	0.40, 2.60	0.957
Infant age (months)	0.98	0.88, 1.10	0.779
Male sex	1.36	0.62, 3.03	0.444
Length for age Z-score	0.96	0.76, 1.22	0.742
f. Multivariate model, continuous IL-6 response to *E. coli*			
IL-6 response to *E. coli**	1.42	1.06, 1.90	0.019
Infant age (months)	0.98	0.88, 1.10	0.786
Male sex	1.42	0.64, 3.14	0.389
Length-for-age Z-score	0.96	0.76, 1.22	0.766
g. Multivariate model, both binary IL-6 responses to stimuli (increase/no increase)			
IL-6 response to *S. enterica*	0.52	0.17, 1.57	0.245
IL-6 response to *E. coli*	1.47	0.47, 4.63	0.508
Infant age (months)	0.98	0.88, 1.10	0.771
Male sex	1.41	0.63, 3.15	0.403
Length for age Z-score	1.00	0.63, 3.15	0.991
d. Multivariate model, both continuous IL-6 responses to stimuli			
IL-6 response to *S. enterica**	0.96	0.71, 1.30	0.776
IL-6 response to *E. coli**	1.44	1.05, 1.99	0.022
Infant age (months)	0.98	0.88, 1.10	0.772
Male sex	1.43	0.64, 3.16	0.383
Length-for-age Z-score	0.98	0.76, 1.26	0.856

**ln*-transformed

23

Table S8. Full CPH models: any infectious disease

	HR	95% CI	p
a. Bivariate models:			
sIgA[*]	0.75	0.48, 1.19	0.221
Baseline IL-6[*]	0.93	0.65, 1.31	0.666
Binary IL-6 response to *S. enterica*	0.39	0.22, 0.68	0.001
Continuous IL-6 response to *S. enterica*[*]	0.86	0.71, 1.05	0.148
Binary IL-6 response to *E. coli*	0.60	0.34, 1.05	0.072
Continuous IL-6 response to *E. coli*[*]	1.13	0.91, 1.42	0.267
b. Multivariate immune content model:			
sIgA[*]	0.88	0.47, 1.66	0.693
Continuous IL-6 response to *S. enterica*[*]	0.78	0.63, 0.96	0.018
Continuous IL-6 response to *E. coli*[*]	1.32	1.02, 1.72	0.037
Infant age (months)	0.97	0.90, 1.04	0.345
c. Multivariate model, binary IL-6 response to *S. enterica* (increase/no increase)			
IL-6 response to *S. enterica*	0.40	0.22, 0.73	0.003
Infant age (months)	0.96	0.90, 1.03	0.249
Male sex	1.42	0.85, 2.36	0.177
Length for age Z-score	1.00	0.86, 1.16	0.990
d. Multivariate model, continuous IL-6 response to *S. enterica*			
IL-6 response to *S. enterica*[*]	0.72	0.55, 0.93	0.012
Infant age (months)	0.94	0.88, 1.00	0.061
Male sex	0.72	0.35, 1.48	0.372
Length for age Z-score	0.95	0.82, 1.11	0.549
Sex*IL-6 response to *S. enterica*	1.59	1.08, 2.34	0.019
e. Multivariate model, binary IL-6 response to *E. coli* (increase/no increase)			
IL-6 response to *E. coli*	0.62	0.35, 1.11	0.111
Infant age (months)	0.98	0.91, 1.06	0.653
Male sex	1.08	0.63, 1.84	0.773
Length for age Z-score	0.98	0.84, 1.13	0.751
f. Multivariate model, continuous IL-6 response to *E. coli*			
IL-6 response to *E. coli*[*]	1.14	0.91, 1.43	0.249
Infant age (months)	0.97	0.90, 1.04	0.372
Male sex	1.14	0.67, 1.96	0.632
Length-for-age Z-score	0.96	0.83, 1.13	0.650
g. Multivariate model, both binary IL-6 responses to stimuli (increase/no increase)			
IL-6 response to *S. enterica*	0.37	0.18, 0.77	0.008
IL-6 response to *E. coli*	2.48	0.70, 8.74	0.157
Infant age (months)	1.09	0.97, 1.23	0.154
Male sex	1.32	0.76, 2.29	0.326
Length for age Z-score	0.99	0.85, 1.16	0.899
LAZ*IL-6 response to *E. coli*	0.85	0.73, 0.99	0.042
d. Multivariate model, both continuous IL-6 responses to stimuli			
IL-6 response to *S. enterica*[*]	0.77	0.62, 0.96	0.019
IL-6 response to *E. coli*[*]	1.30	1.03, 1.65	0.030
Infant age (months)	0.96	0.89, 1.03	0.265
Male sex	1.14	0.67, 1.95	0.628
Length-for-age Z-score	1.00	0.86, 1.17	0.959

[*]*ln*-transformed
